# Percolation networks inside 3D model of the mineralized collagen fibril

**DOI:** 10.1038/s41598-021-90916-x

**Published:** 2021-05-31

**Authors:** Fabiano Bini, Andrada Pica, Andrea Marinozzi, Franco Marinozzi

**Affiliations:** 1 Department of Mechanical and Aerospace Engineering, “Sapienza” University of Rome, via Eudossiana, 18 - 00184 Rome, Italy; 2 Orthopedy and Traumatology Area, “Campus Bio-Medico” University, via Alvaro del Portillo, 200 - 00128 Rome, Italy

**Keywords:** Biomedical engineering, Orthopaedics

## Abstract

Bone is a hierarchical biological material, characterized at the nanoscale by a recurring structure mainly composed of apatite mineral and collagen, i.e. the mineralized collagen fibril (MCF). Although the architecture of the MCF was extensively investigated by experimental and computational studies, it still represents a topic of debate. In this work, we developed a 3D continuum model of the mineral phase in the framework of percolation theory, that describes the transition from isolated to spanning cluster of connected platelets. Using Monte Carlo technique, we computed overall 120 × 10^6^ iterations and investigated the formation of spanning networks of apatite minerals. We computed the percolation probability for different mineral volume fractions characteristic of human bone tissue. The findings highlight that the percolation threshold occurs at lower volume fractions for spanning clusters in the width direction with respect to the critical mineral volume fractions that characterize the percolation transition in the thickness and length directions. The formation of spanning clusters of minerals represents a condition of instability for the MCF, as it could be the onset of a high susceptibility to fracture. The 3D computational model developed in this study provides new, complementary insights to the experimental investigations concerning human MCF.

## Introduction

Natural structures present complex hierarchical architectures that represent optimized solutions achieved during the evolutionary process for a given set of requirements and constraints. Many biological materials must be simultaneously light weight, strong, flexible and resilient. To offer similar outstanding mechanical properties, that often surpass those of their components by orders of magnitude, the approach observed in nature is a combination of stiff and soft components in hierarchical structures, as it is the case of bone^1^.

Bone is a natural composite with a complex multiscale arrangement of structures characterized by up to 12 nested levels of organization, from macro- to nanoscale^2^. The spatial organization at every length scale influences the material behaviour, leading to radically different material characteristics in order to meet specific functions^1^. Specifically, at the nanoscale, the structural unit of bone tissue, i.e. the mineralized collagen fibril (MCF), is characterized by a combination of type I collagen, apatite mineral, water and non-collagenous proteins (NCPs)^3,4^. The collagen component provides mechanical stability and elasticity to the structure^5^, while the brittle apatite minerals increase stiffness, fracture strength and robustness of MCF^1,6^. The spatial organization of apatite crystals and collagen matrix is a topic of intense research since changes in bone mechanical properties and alterations of its functions are often correlated with abnormal variations at the nanostructure^6^.

Collagen molecules self-assemble in a staggered manner into fibrils with diameters ranging from 50 to 200 nm and length of about 1000 nm^6,7^. Apatite crystals are located both within and between collagen fibrils, i.e. intra- and interfibrillar mineralization respectively^8–13^. A widely accepted model of the 3D arrangement of apatite minerals within the MCF assumes that in the longitudinal direction mineral crystals are disposed in a staggered arrangement, while parallel layers of apatite span the equatorial plane of the fibril^14^. According to^15,16^, the architecture of the collagen phase as an ordered matrix contributes to the co-alignment between the crystallographic *c-axis* of apatite and the longitudinal axis of collagen fibril with an angular distribution of ± 20 degrees^15,17^.

To assess the composition and structure of bone, several techniques concerning the tissue characterization have been developed. X-ray microtomographic technique determines bone structural organization at the macro- and micro-scale^18–20^. However, information about mineral morphology and orientation is still elusive, as nanometre resolution and a large field of view up to the micrometre scale are required to investigate them. To date, observations based on transmission electron microscopy^21^ and small-angle X-Ray scattering technique^6^ allow to define apatite minerals as mainly platelet-like shaped with low dispersion in thickness, i.e. 2–5 nm, but wider spread in length (50–170 nm) and width (5–90 nm). The combination of advanced analysis methods^22^ also showed that the individual apatite platelets are encapsulated in a core–shell structure by an additional non apatitic environment, i.e. hydrated amorphous calcium phosphate layer that provides a favourable chemical environment for the interaction between mineral platelets.

From tomographic data^2,16^ it emerged also the evidence that the mineral phase is present as individual crystallites and as aggregates. Recent investigations of Xu et al.^16^ at the length scale of a single fibril identified, on average, small stacks of 2–4 platelets from the analysis of 300 nm long MCF with a diameter of 100 nm, that corresponds to approximately 150 platelets. Conversely, in areas with higher mineral density, larger groups of roughly 8 platelets were individuated^16^. Landis et al.^17^ suggested that apatite crystal fusion can influence the biomechanical properties of bone.

In addition to experimental investigations^17–21,23,24^, computational techniques based on probabilistic methods^25,26^, molecular dynamics simulations^5,27,28^, finite element methods (FEM)^29–31^ and homogenization theory^32–36^ have been developed to assess the contribution of the nanoscale components to the mechanical performance of bone. Depalle and co-workers^27^ highlight from atomistic simulations that MCF reaches its optimal properties in terms of toughness and strength for a mineral density around 30%. Furthermore, from the work of Fielder and Nair^28^ it emerged that the effect of water variation on MCF stiffness is not statistically significant. Thus, compared to water content, apatite mineral plays a dominating role in affecting the mechanical deformation.

Bone ultrastructure has been investigated also by means of mathematical models based on continuum micromechanics and homogenization theory in the studies of Hellmich and colleagues^32–36^. Several models with increasing complexity have been developed. Initially a two-phase isotropic crystal foam composed of a hydroxyapatite phase and a nonminerally phase was considered^32^. Subsequently, it was assumed an interpenetrating network of apatite crystals and collagen molecules^33^, while the third model considered composite of fibrils, i.e. collagen apatite networks embedded in a collagen-free extrafibrillar mineral foam matrix^34^. The models predicted the elastic properties of bone as a function of mineral volume fraction in good agreement with large experimental database^37,38^. It is noteworthy that the structural representation of bone ultrastructure developed in these models^32–36^ did not take into account the spatial organization of the apatite minerals. Conversely, FEM studies of Vercher-Martinez^29^ have outlined that the arrangement of apatite minerals, the distance between apatite crystals and the mineral overlapping have considerable influence on the mechanical behaviour of the MCF. Moreover, investigations based on FEM attempted to get deeper insight into the role of the nanocomponents by considering also sub-structures of the MCF, i.e. collagen microfibril^30,31^, or organic constituents as collagen crosslinks^30^.

Therefore, computational methods analysed the properties of bone at several length scales in function of the mineral volume fraction, apatite aspect ratio, collagen-apatite 3D arrangement and the presence of further complexities of the structure, e.g. water content, NCPs or collagen crosslinks^30,31^. As illustrated from several perspectives, the organization and interactions of the apatite minerals at the nanometre scale have relevant impact on the functionality of bone tissue. It is worth noticing that insufficient resolution in the case of experimental studies and high computational costs of numerical simulations have limited the analysis to reduced regions of the MCF.

Moreover, although recent investigations^2,16^ have provided insights into the 3D arrangement of the mineral within the MCF, the kinetics related to nucleation and growth of apatite platelets are still scarcely studied in literature despite their importance in governing the structural properties of MCF. Apatite growth can involve two main processes: addition of monomeric chemical species on existing crystals or attachment of nanoparticles. Experimental studies^39^ suggest that nanocrystals attachment achieved through collision and coalescence events are prevalent.

The mineralization of bone nanostructure is a complex process that influences the structural and functional characteristics of bone. The presence of apatite crystallites aggregates remains intriguing since visualization of minerals at bone ultrastructural level is still challenging. This study aims to explore, using the percolation theory, whether the apatite minerals may form connected network at bone nanoscale level.

Percolation theory presents an interesting paradigm to investigate scenarios of MCF mineralization since it investigates the appearance, evolution and properties of connected elements^40^. In a physical system, the onset of a long range connected component is characterized by a transition from containing groups of isolated particles to a connected network. A percolation phenomenon is represented by a phase transition that leads to changes in the mechanical, physical and chemical characteristics of the material. For instance, the emergence of connected clusters may modify the conductivity processes, the kinetics of chemical reactions, the mechanical toughness of a material. Consequently, the most studied aspects of percolation phenomena are those in proximity to the onset of global connectivity, i.e. close to the percolation threshold.

Continuum percolation is a more realistic model of percolation process that occurs in heterogeneous materials. In continuum percolation, the system is composed of objects that are randomly placed in space. The elements may have various sizes and shapes and, if non spherical, also a distribution of their orientations is considered^40^. Continuum percolation studies have become increasingly frequent in literature since they generate a bridge between the mathematical approach and properties of lattice percolation and the realistic models of heterogeneous materials, namely porous media, composite materials or colloids^40^. Typical applications of the continuum percolation method consider electrical conductivity of carbon nanotubes, rheological properties such as permeability and related phenomena, i.e. diffusion, drainage in porous media. It was also used for the study of fracture network in continuous systems, the analysis of chemical and biological networks and to get insight into the mechanical properties of articular cartilage^41^. Associated with bone tissue, percolation features are mentioned in the analysis of mechanical properties of partially mineralized tissue, which is found at the attachment of tendon to bone insertion^42^. Moreover, in micromechanics models studying bone ultrastructure^32–36^, the bistable mechanical behaviour of the tissue in function of mineral volume fraction is described in terms of percolation threshold. It is worth pointing out that these previous works^32–36,42^ did not attempt to identify the formation of percolating paths.

In this study, we aim to investigate whether the mineral phase exhibits percolation-like characteristics according to^40^, given the similarities, between the bone mineralization process and percolation phenomena in terms of transition from stable to unstable system depending on system volume fraction. Briefly, this study attempts to explore if in correspondence to high mineral content, the increase of apatite crystal sizes may be also associated with the aggregation of mineral platelets.

In this work, we reduce the complexity of the MCF structure by considering an idealised continuum 3D model of apatite minerals within the MCF. However, we have taken into account several elements in order to better mimic the real structure of bone tissue at nanoscale. It is worth mentioning that most of 3D continuum percolation studies maintain constant dimensions of the particles within the simulation box. Conversely, we assumed variable values for the geometric sizes of apatite crystals, in agreement with experimental observations^21^. Moreover, differently from percolation models available in literature that consider cubic simulation box, in this model we assumed a cylindrical box that better mimics the real shape of the MCF.

The representation of the underlying atomic structure is beyond the scope of this work. The apatite minerals represent an ideal target to apply the concepts of percolation theory. In fact, the inclination of the platelets in the interval ± 20 degrees leads to slightly disordered configurations, in agreement with the initial assumptions of the percolation theory that defines a percolation cluster as a network of disordered elements. Conversely, the collagen molecules are aligned in the longitudinal direction of the MCF describing an ordered organic matrix^7^. Therefore, it is considered compelling the development of a specific numerical algorithm that assesses the probability of a percolation-like behaviour for apatite minerals. In this initial study, we omit the presence of water, NCPs and collagen crosslinks since their role is mostly linked with a mechanical response of the MCF, which is beyond our present scope.

## Results

We applied the continuum percolation theory^40^ to assess the probability of formation of long-range connected networks within the MCF. In order to investigate the scenario characterized by apatite crystals aggregation^43^, we implemented a 3D model assuming that apatite platelets can interpenetrate each another for up to 10% of apatite platelet volume. We have performed Monte Carlo simulations (Mathematica 11, Wolfram, Oxfordshire, UK) to investigate the presence of connected networks for ten values of mineral volume fraction (VF), ranging from 7 to 52% and for two MCF diameters, namely 50 nm and 200 nm^2,3,6^.

**Figure 1:**
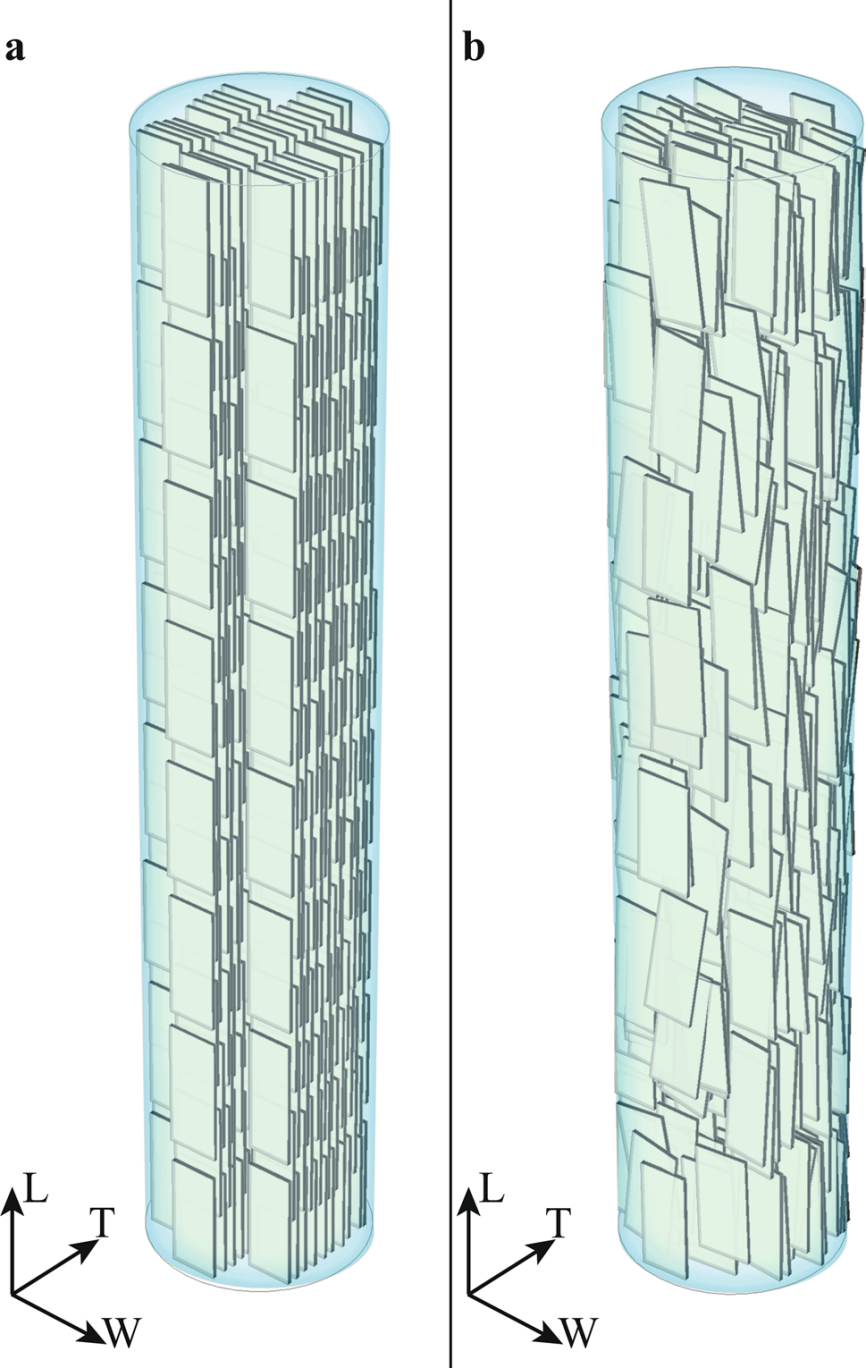
3D Representation of the mineralized collagen fibril (light blue). In (**a**) starting configuration of apatite minerals (grey), aligned with the axes of the coordinate system. In (**b**) configuration of mineral phase after roughly 2.1 × 10^6^ moves and rotations.

Computer simulations of systems that undergo percolation phenomena is a two-step process. Firstly, for each mineral VF, we generate different configurations of the geometrical model using the Metropolis algorithm^40^, in which platelets start in a regular configuration and then are subjected to random displacements and inclinations (Fig. 1). Each perturbation is accepted whether do not cause an interpenetration superior to 10% apatite crystal volume^40^. We considered overall up to 60 × 10^6^ displacements and rotations for each MCF diameter. Secondly, for each configuration we verified whether connected networks are present.

The configurations obtained after the equilibrium are used to perform cluster analysis. A connectivity criterion is required to determine whether the platelets reside within the same cluster or not. Firstly, we investigate the connectivity related to the mutual attraction between minerals, which occurs via their hydrated layers^22^. The establishment of long-range electrostatic interactions is modelled considering a soft shell of thickness δ/2 that covers the nanocrystals. Two adjacent crystals belong to the same cluster if their minimum distance is smaller than δ. We considered a value of δ = 14 Å, that corresponds to the cut off distance of long-range electrostatic interactions between apatite crystals^22^. Secondly, we introduce a more restrictive condition by assuming that two apatite platelets are connected if they interpenetrate each other. This scenario represents groups of minerals that coalesced and form a continuum.

A system percolates if a cluster spans the domain in a specific direction from one boundary to the opposite one^40^. We determined whether a cluster is percolating or not by means of an algorithm that for a given cluster compares the position of the platelets that compose it with the boundaries of the MCF.

The percolation probability for each mineral VF is obtained as the ratio between the number of configurations where a percolating cluster appeared and the number of realizations assessed by the cluster analysis. The resulting probabilities were plotted in function of the mineral volume fraction and fitted with a hyperbolic tangent function^40^ (Eq. 10).

**Figure 2:**
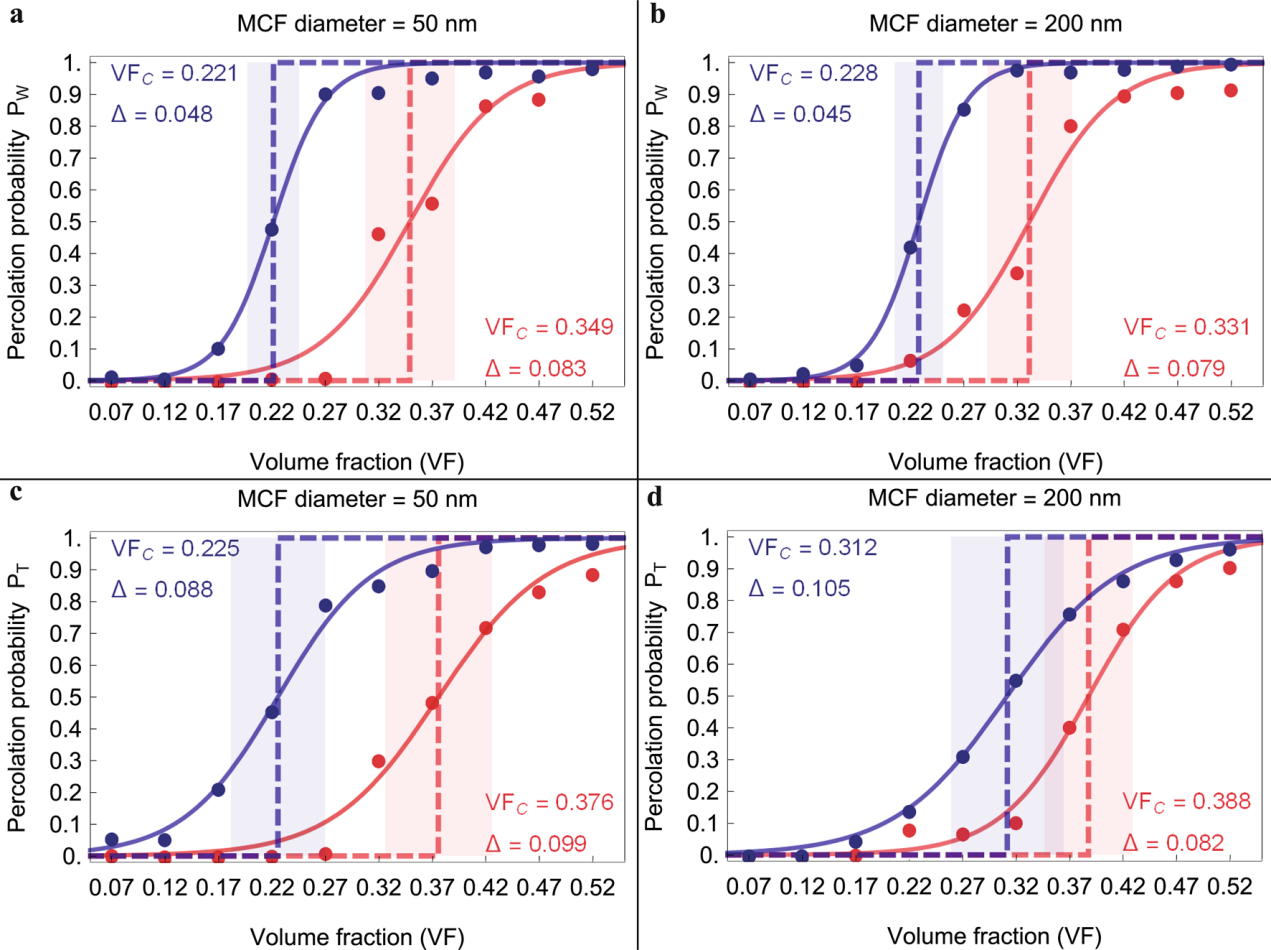
Percolation probability as a function of mineral volume fraction for spanning clusters in W (**a**, **b**) and T (**c**, **d**) direction. The percolation probability is calculated for clusters achieved assuming long-range interaction between minerals (blue) and interpenetrating crystals (red). VF_c_ represents the critical VF at which the percolation probability is equal to 0.5 and Δ characterizes the width of the percolation transition.

**Figure 3:**
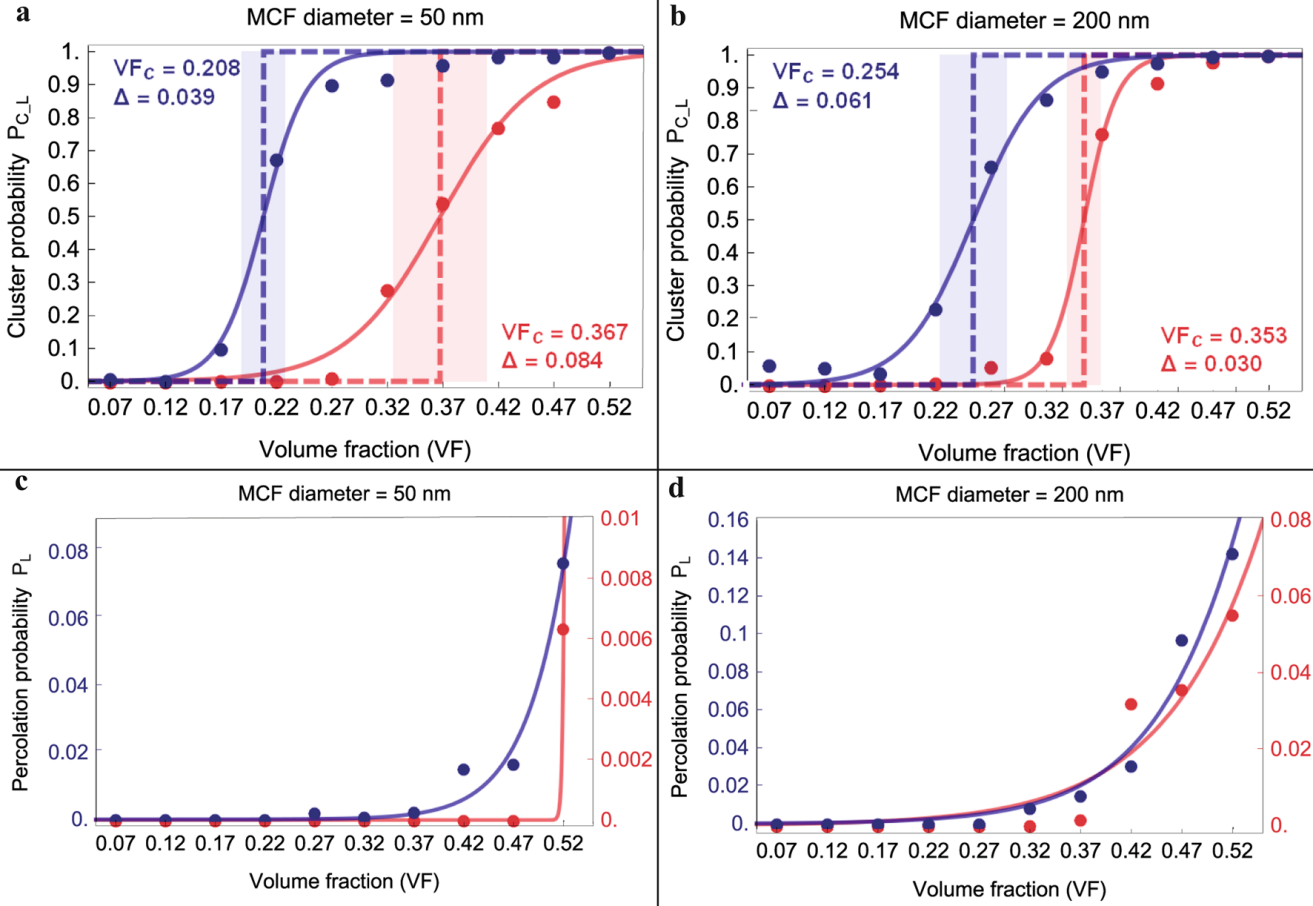
Cluster formation probability in longitudinal direction for fibril with reduced length according to experimental data^2^ (**a**, **b**). Percolation probability calculated for clusters that span the entire length of mineralized collagen fibril (**c**, **d**). Clusters achieved assuming long-range interaction between minerals (blue) and interpenetrating crystals (red) were considered. VF_c_ represents the critical VF at which the percolation probability is equal to 0.5 and Δ characterizes the width of the percolation transition.

Percolation theory allows to identify a critical VF, i.e. the percolation threshold, at which the system passes from having isolated clusters to connected networks. According to^40^, we assume that the percolation threshold is determined by the mineral VF corresponding to a percolation probability of 0.5. In Figs. 2 and 3 we represent the percolation probability as a function of mineral VFs for two different MCF diameters.

Experimental studies showed that the spatial arrangement of the minerals is highly anisotropic^3,15^. In order to observe the effect of anisotropy on the percolation probability, we analysed separately the spanning clusters in the width (Fig. 2a,b), thickness (Fig. 2c,d) and longitudinal direction (Fig. 3) of the MCF, respectively. Furthermore, the percolation network in the longitudinal direction is evaluated for different MCF lengths. We considered reduced MCF lengths, following the experimental observations of Reznikov et al.^2^, and the average length of MCF reported in Literature^5,6^, which is roughly 1000 nm.

Moreover, for each direction and for each MCF diameter, we present two sets of percolation curves that describe the percolation probability in function of the adopted connectivity condition.

## Discussions

The arrangement of apatite mineral at the nanoscale has been the object of experimental studies^2,3,8–13,15–17,43–46^ and computational models^25–36^. The mineral matrix represents one of the major players in determining the mechanical properties of bone tissue. In this study, we analysed possible implications in apatite crystals arrangement due to a variation of the mineral content.

Firstly, the results depicted in Figs. 2 and 3 show that the extent of the apatite network and the percolation threshold depend on the connectivity distance. Lower critical VFs are obtained applying the connectivity criterion based on the cut off distance of long-range interactions, than the critical VFs achieved by considering interpenetrating platelets. This behaviour is predicted also by an analytical relation (see Eq. S15 in Supplementary Information) which assumes that the critical VF is inversely proportional to the volume of the connecting region around a platelet.

Furthermore, we observed that the anisotropy of MCF determines different percolation behaviours. In the equatorial plane (Fig. 2), for both MCF diameters, the percolation transition is characterized by lower mineral VF for clusters spanning the system in W direction, with respect to the percolating clusters in T direction. Namely, along the W direction, the probability of formation of percolating clusters composed of interpenetrating platelets rises sharply for VF greater than 35% for MCF of 50 nm diameter and 33% for MCF of 200 nm diameter. Spanning clusters of platelets connected via their hydrated layer show a high probability of formation in W direction for VF greater than 22% for both MCF diameters. In T direction, a steep increase of the probability of percolating clusters composed by interpenetrating apatite minerals is observed for VF greater than 37% and 38% for MCF diameter of 50 nm and 200 nm, respectively. The spanning clusters with platelets connected via their hydrated layer are characterized in T direction by an increased probability for VF greater than 22% in the MCF with 50 nm diameter and VF higher than 31% for MCF of 200 nm diameter.

In the longitudinal direction, considering reduced lengths of the MCF (Fig. 3a,b), the probability of individuating spanning clusters rises for VF greater than 20% for platelets connected by means of their hydrated layers, while for interpenetrating platelets, the percolation probability increases for VF greater than 35%. In the case of average full length of MCF, there is a low probability to develop spanning clusters of interpenetrating platelets. The highest percolation probability in longitudinal direction (Fig. 3c,d), is inferior to 10% for both MCF diameters, while in the equatorial plane, the maximum percolation probability is roughly 1. Conversely, the spanning clusters in L direction characterized by the connectivity condition related to the hydrated layers of minerals have slightly higher percolation probabilities.

**Figure 4:**
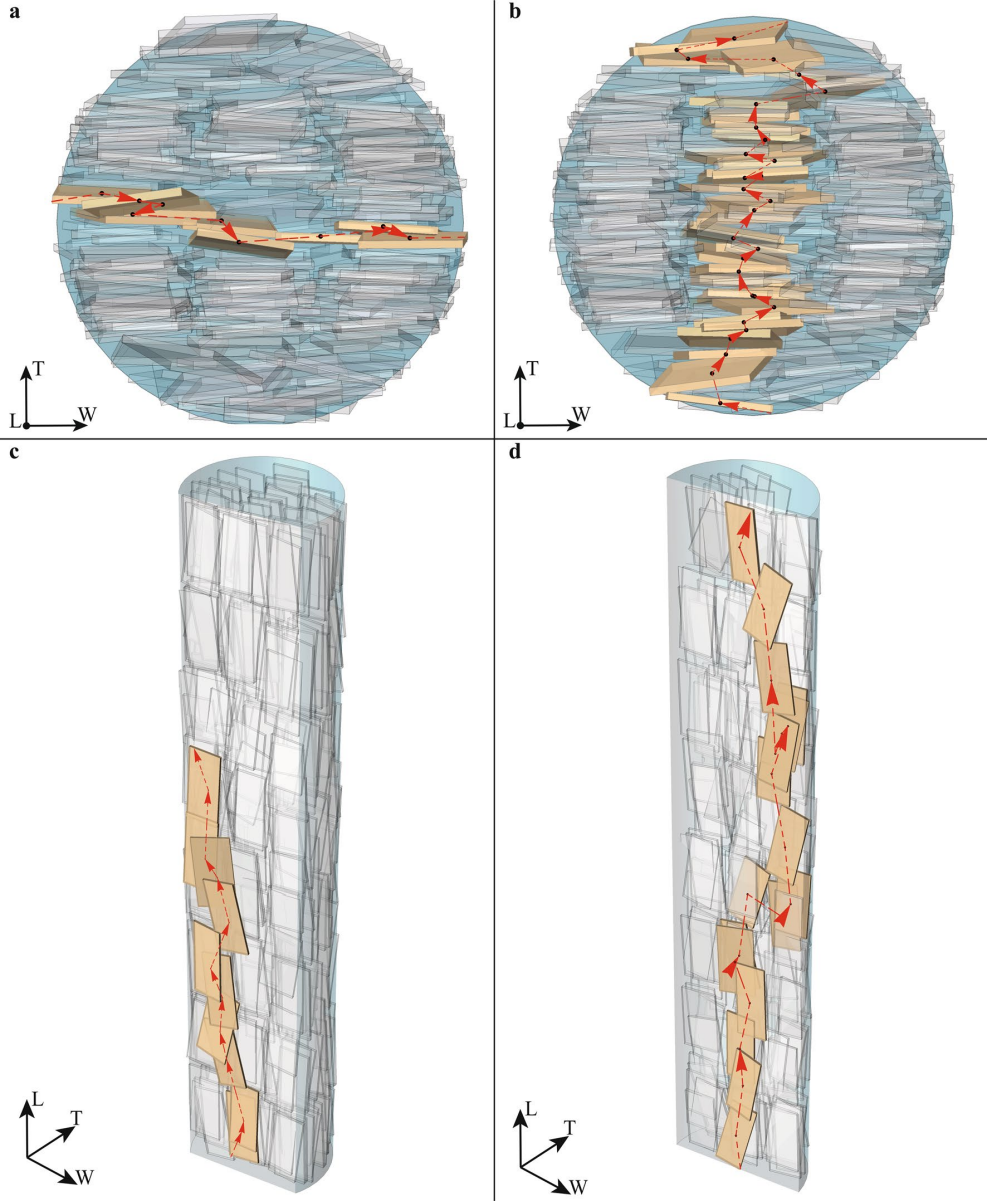
In (**a**,**b**) percolating clusters in W and T direction for the mineralized collagen fibril with diameter of 200 nm. In (**c**) cluster with reduced length according to experimental data^2^, in (**d**) spanning cluster along the fibril length.

The curve of percolation probability is associated with the structural changes subsequent to increasing mineral VF. The percolation threshold separates the system into two regions: below the critical VF, the MCF is characterized by incomplete and less extended networks of apatite crystals, while above the percolation line, the system has spanning networks of connected platelets (Fig. 4). The findings of the percolation model supported our hypothesis and demonstrated that increased mineral VF leads to the onset of spanning connected networks of mineral crystals. Therefore, the results highlighted that MCF is characterized by a percolation-like behaviour.

The findings are in good agreement also with the recent experimental study^16^ that observed the existence of aggregated mineral crystals in groups up to 8 platelets within the MCF.

It is worth pointing out that the results presented in the study of Xu et al.^16^ are within the limitations of the high-resolution electron tomography technique applied. The MCF region investigated experimentally is characterized by a length of 300 nm and a diameter of 100 nm, encompassing approximately 150 platelets. Conversely, the geometric model proposed in this study considers MCF of 1000 nm length and 50 nm respectively 200 nm in diameter, in agreement with^3,5,6^. In this perspective, the computational 3D model developed in this study provides new, complementary insights to the experimental assessment performed in^16^. Consequently, the development of clusters with a higher number of platelets than the stacks detected by Xu et al.^16^, e.g. spanning cluster in T direction (Fig. 4b), is justified by the enlarged dimensions of the nanostructure, i.e. the entire MCF, and by the wide range of mineral VFs considered in the present model.

The MCF mineralization is a fundamental process and the biofeedback system represents the key to the structural and functional success of bone tissue. Crystal nucleation and growth are finely regulated by the process of bone remodelling, which is an example of mechanoregulation based on a feedback loop^47^. In healthy conditions, the structural adaptation to the mechanical stimulus is obtained through a dynamic equilibrium between bone formation governed by osteoblasts and bone resorption directed by osteoclasts. An imbalance in the rate of bone turnover leads to an instability of the system that might compromise tissue functionality^47^. For instance, if remodelling rate decreases, older hypermineralized regions are not replaced by new hypomineralized structures. Consequently, a high degree of mineralization induces residual strains to neighbouring mineral crystals and collagen molecules, increasing the susceptibility of fracture^48^. Impairment of bone remodelling rate may lead to the increase of apatite crystals sizes and subsequently to higher mineral content^49^.

The authors believe that the dynamical equilibrium that describes the bone remodelling process is detectable also from the percolation curve. Below the critical mineral VF, the low percolation probability indicates that the MCF is characterized by isolated clusters in line with experimental observation of bone samples that represent physiological conditions of the tissue^16^. Above the percolation threshold, the MCF is characterized by spanning clusters of connected apatite platelets that are related to anomalies in the remodelling process. According to experimental investigations, increased crystal size and mineral aggregation are consequences of abnormal turnover rate due to aging, disuse or hormonal alterations^48–50^.

The onset of percolating clusters may conceivably introduce changes in the mechanical, physical and chemical characteristics of the nanostructure^17^. Currey^51^ suggested that the increase of mineral content may lead to a fusion of apatite platelets. The application of the percolation theory and the identification of clusters of interpenetrating apatite platelets allow to better highlight this hypothesis. In addition, the critical VF corresponding to clusters composed of platelets that are connected by means of their hydrated layers may represent the transition from a MCF with features controlled by the collagen phase to a MCF with properties controlled by the mineral content^42^.

Bone nanostructure should be strong and lightweight to facilitate movement. From experimental investigations^2,16^ and atomistic models^27^ it emerged the idea that exists an optimized limit to apatite crystal size and to the amount of mineral within the MCF that is a prerequisite for normal bone structure and function. Computational models^27^ highlighted that an optimized environment where the individual constituents perform their normal function is characterized by a mineral content around 30%. It is worth pointing out that the findings achieved applying the percolation theory are in line with these studies. Namely, considering percolating clusters composed of interpenetrating mineral platelets, the critical VF is slightly higher than 30% for both MCF diameters analysed. Hence, a MCF characterized by a mineral content of roughly 30% represents a stable system, with isolated clusters.

These findings are encouraging for the improvement of the design of biomaterials. In fact, a significant challenge for the development of biomimetic composite substitute with mineral constituents involves providing tissue specific characteristics. The functionality of the regenerated tissue depends on the micro- and nano-structure of the 3D scaffold. The latter should possess interconnected pores that support vascularization, transport of nutrients and metabolic waste as well as maintaining the mechanical features of strength and toughness^52^. The percolation phenomenon associated with the apatite mineral content within MCF leads to a bistable behaviour that influences bone tissue properties. Avoiding mineral volume fractions near the percolation threshold allows to modulate with higher accuracy the mechanical and biological performance of the scaffold. Information concerning the number of apatite platelets and their arrangement helps the effective biomimicry of the physiological nanostructure and may enable a higher control over the final properties of the scaffold.

As a final note, we want to highlight some limitations of our study. A common difficulty in modelling biological tissues is the lack of shape uniformity among elements. The geometry observed in real tissues is much more complex than the geometrical model proposed here on many aspects. We assumed ideal plate-like shaped apatite crystals, according to^3,17^. It is worth mentioning that recently Reznikov et al.^2^ observed mineral particles both as needle- and platelet-like shaped. However, the X-Ray diffraction-based investigations of Xu et al.^16^ emphasized that bone mineral principally comprises platelets-like apatite crystals rather than needle-like crystals.

Given the limited evidence in literature concerning apatite mineral aggregation, in our model crystal coalescence is represented by an interpenetration up to 10% of mineral volume. Despite these simplifications, we achieve reasonable evidence concerning the percolation-like behaviour of apatite minerals within the MCF. Moreover, in this first computational model, we do not focus on the interactions between the mineral array and the other nanoscale components, i.e. collagen matrix, crosslinks and NCPs. The aforementioned simplifying assumption is also corroborated by TEM observations^16,17^. These studies suggest that the collagen matrix is flexible enough to handle crystal aggregation. Therefore, apatite minerals may push the collagen aside and develop into a preferred morphology^16^. The role of NCPs in collagen mineralization is still a subject of debate^30,32,53^. Several studies^4,46^ have outlined that these proteins play a significant role in interfibrillar mineralization while available data concerning the effect on intrafibrillar mineralization is limited. Experimental investigations^53^ show that NCPs has a significant contribution to the mechanical behaviour of the MCF, which is out of the scope of the present work.

In addition, limitations concerning the code implementation are reported in Supplementary Information (see Sections S2, S4).

In this study, we investigated the feasibility that a percolation network composed of brittle apatite crystals may be formed at bone nanoscale. Furthermore, we achieved the critical mineral VFs at which percolation transition occurs for the three main spatial directions, i.e. width, thickness and length direction. According to the percolation theory^40,54^, the onset of connected clusters preludes to changes in the mechanical response of a material. When analysing damage and failure, the percolation threshold represents the point at which an initial intact sample suddenly develops a fracture in response to stress^55^. From this point of view, the findings of this study suggest the necessity of further investigations aimed at understanding the role and influence of connected networks of apatite minerals in the development of preferential sites to nanocrack propagation. In future studies, to better evaluate the mechanical effects of apatite clusters on MCF behaviour will be necessary to consider the contribution and spatial distribution of all nanocomponents of the fibril, including collagen crosslinks and NCPs.

In conclusion, the percolation transition that occurs at high VF for spanning clusters of interpenetrating platelets may provide insights into the onset of spontaneous fracture, often related to pathological conditions like osteoporosis. As documented by experimental studies^48,56^, osteoporotic tissue is characterized by increased mineral content and microcrack density. Our findings showed that in conditions of pharmaceutical osteoporotic treatment, i.e. VF greater than 37%, the MCF is characterized by a high probability of percolation that in turn is indicative of high susceptibility to spontaneous fracture.

## Methods

### Model parameters

The MCF is represented by a cylinder, which dimensions are consistent with the morphological characterization of the fibril available in literature^2–6^. Human MCFs typically have a diameter^6^ between 50 and 200 nm and length^5^ in the order of 1000 nm. We developed models for two different MCF diameters, namely 50 nm and 200 nm.

Apatite crystals are described as thin elongated platelets^21^. We represented them as parallelepipeds with variable geometric dimensions. The crystals organization within the MCF is defined by a staggered arrangement in the longitudinal direction of the collagen fibril and parallel layers in the equatorial plane^14,44^ (Fig. 1a). For each MCF diameter, we analysed ten different mineral volume fractions: from low (7%) to high (52%) mineralization degrees with steps of 5% (see Supplementary Information).

### Generating realizations

For each mineral content of the MCF, we used the Metropolis algorithm^40^ to generate mineral configurations. Initially, the centroids of the apatite platelets are placed in correspondence of the sites of a staggered prismatic lattice within the cylinder (see Supplementary Information). The length, width and thickness axes of the platelets are initially perfectly aligned with the axes L, W and T of a global coordinate system (CS). The MCF equatorial plane is identified by W and T axes.

We initialize the mineral configuration by occupying the lattice sites randomly. Once the platelets are arranged, each mineral is moved by displacing it along each axis by random amounts chosen from a uniform distribution in the interval [− τ; τ], where τ is the maximum step size^40^. Subsequently, the platelet is rotated around each axis by amounts extracted from a Gaussian probability distribution function with mean 0 degrees and standard deviation θ, where θ is the maximum inclination size. The new perturbed position of the platelet is accepted if it does not cause an interpenetration superior than 10% of apatite platelet volume or if it does not go outside the tolerance cylinder. We implemented an interpenetration detection algorithm adapting the geometrical method of Lin et al.^57^ (see Supplementary Information). We set a tolerance cylinder with dimensions 5% greater than the MCF cylinder to allow peripheral platelets to be rotated in the range reported in Literature^15,17^. Otherwise, the move is rejected and the actual position of the mineral is maintained. All platelets are in turn moved according to these criteria. One Monte Carlo cycle, or realization, involves trial translation and rotation around the three CS axes, for all mineral platelets within the MCF model.

The maximum translational and rotational perturbations, i.e. τ and θ respectively, are adjusted at every Monte Carlo cycle to maintain the acceptance ratio of trial moves and rotations close to 0.5^40^. If at the end of a Monte Carlo cycle the acceptance ratio is greater than 50%, τ and θ are incremented by 10% of their actual value, otherwise these parameters are decreased by 10% of their actual value. The updated values are used in the successive Monte Carlo cycle.

### Cluster analysis

After the equilibration (see Supplementary Information), that required overall 2.1 × 10^6^ displacements and rotations per platelets, we performed overall 4.2 × 10^6^ displacements and rotations per platelets for each mineral VF within the MCF of diameter 50 nm and 200 nm, respectively, to analyse the development of clusters. A cluster is formed by a contiguous sequence of pairwise connected platelets^40^. The algorithm that determines the clusters within the MCF is adapted from the tree-based union find algorithm proposed by Newman and Ziff^58^ and it is composed of two main steps: (a) detection of the pairs of platelets that respect a connectivity criterion and (b) identification of all mineral crystals that form a cluster.

The connectivity criterion is based on the minimum distance d between two platelets as obtained from the minimisation problem (Eq. 1):

1 $$ d = Min\sqrt {\left( {w_{i} - w_{j} } \right)^{2} + \left( {t_{i} - t_{j} } \right)^{2} + \left( {\ell_{i} - \ell_{j} } \right)^{2} } $$

where (w_i_, t_i_, *ℓ*_i_) are the coordinates of point p_i_ which is a point of platelet A_i_ and (w_j_, t_j_, *ℓ*_j_) are the coordinates of p_j_ which is a point of platelet A_j_. The equation is solved by means of a gradient based method that minimizes the Euclidean norm between p_i_ and p_j_.

The minimum distance should be determined between each platelet and every other platelet in the MCF. To increase the algorithm efficiency, we calculate only the distances between a reference platelet and the neighbouring mineral crystals which centroids are confined in an exploring volume^40^. The latter has the same dimensions as the exploring volume defined for the algorithm concerning platelets interpenetration.

**Figure 5:**
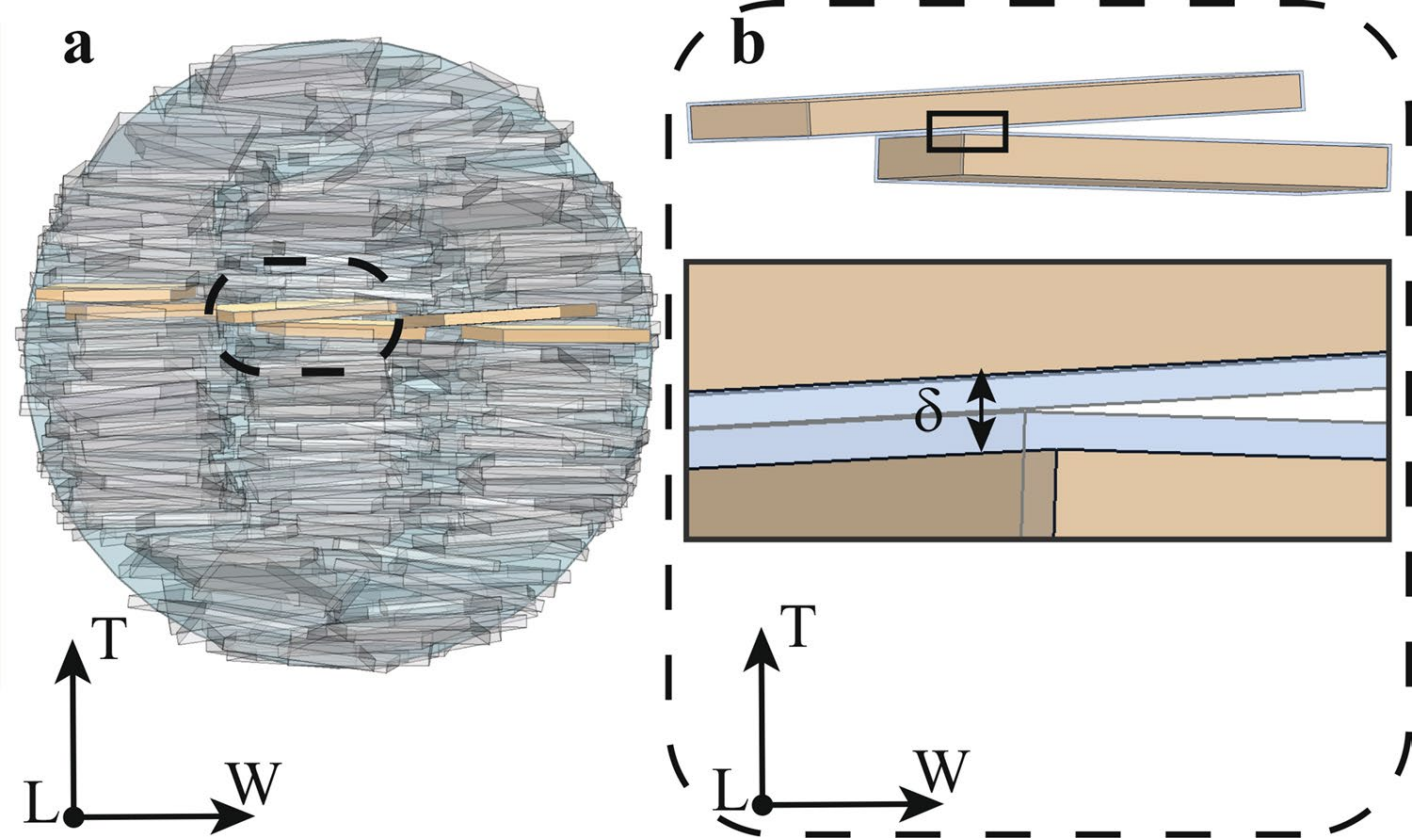
Illustration of a percolating cluster in W direction composed of platelets that verify the cut off distance (δ) of long-range interactions between minerals (**a**). In (**b**) enlarged view of two mineral platelets covered by a shell of thickness δ/2 that mimics the long-range interactions which occur between apatite crystals due to their hydrated layers. The example is achieved from a configuration of apatite mineral at 47% of mineral volume fraction within MCF of 200 nm diameter.

Two platelets are connected if their shortest distance is smaller than a threshold value. In this study, we considered two different values of the threshold. Firstly, we assumed a value that corresponds to the cut off distance of long-range electrostatic interactions between apatite crystals^22^, i.e. δ = 14 Å (Fig. 5).

Thus, two platelets are connected if the following relation is valid:

2 $$ 0 < d \le \delta $$

We assumed that this condition identifies connected platelets by means of their hydrated layers^59^.

Secondly, we analysed the same apatite configuration by considering a more restrictive connectivity condition (Eq. 3):

3 $$ d = 0 $$

**Figure 6:**
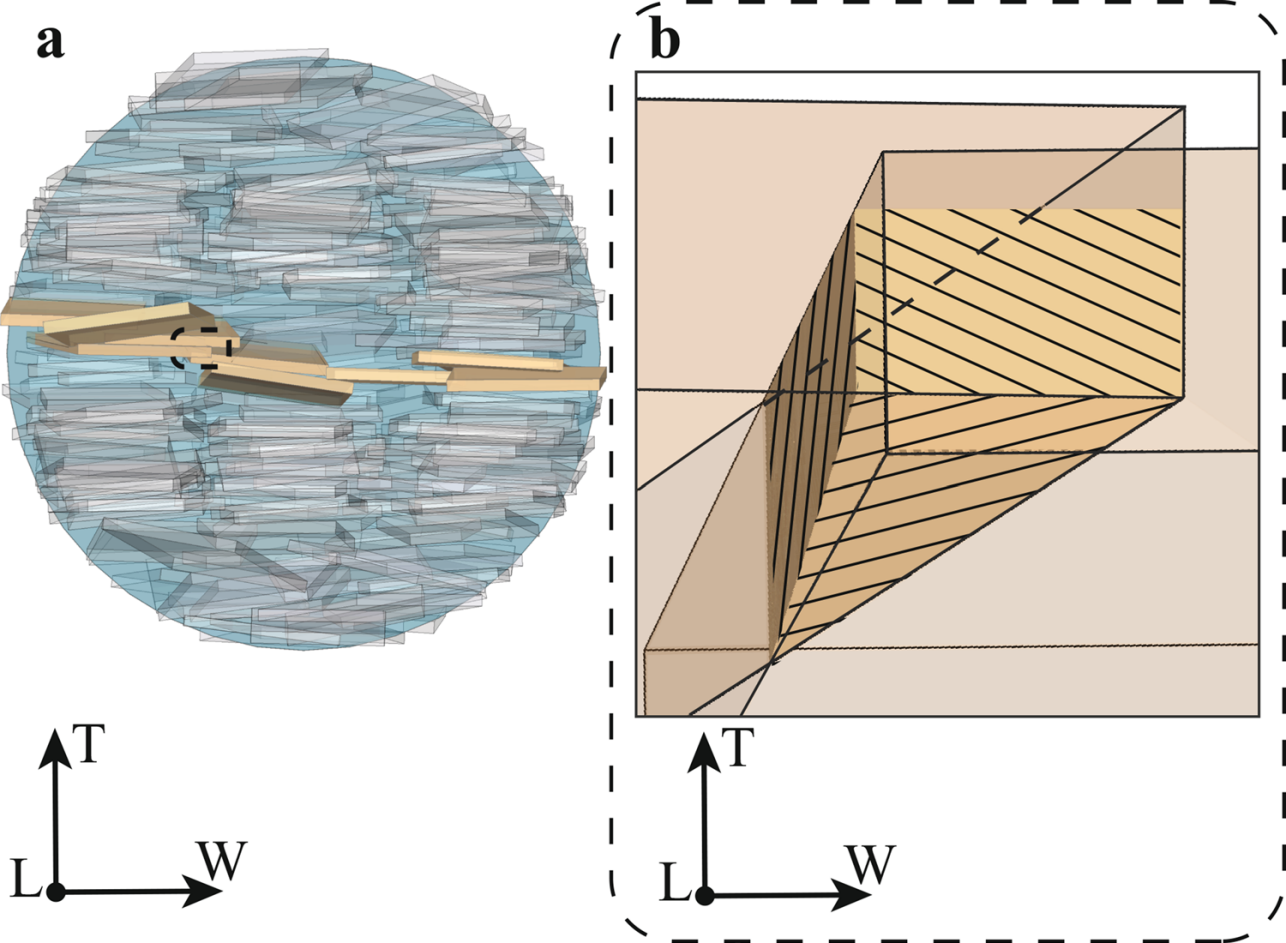
Illustration of a percolating cluster in W direction composed of interpenetrating platelets (**a**). In (**b**) enlarged view of the interpenetration region (hatching filled). The example is achieved from a configuration of apatite mineral at 47% of mineral volume fraction within MCF of 200 nm diameter.

This condition defines as connected only interpenetrating platelets, i.e. mineral platelets form a continuum. In the interpenetration case, the minimum distance is null since the points p_i_ and p_j_ identified by the minimum distance algorithm coincide (Fig. 6).

The pairwise platelets identified for each connectivity condition represent the input data for the cluster identification algorithm, that uses a data tree structure.

Each platelet of the MCF is indexed and it is considered as the vertex of a graph. The pairwise platelets represent the endpoints of the graph edges. Applying the union/find algorithm^58^ for each configuration, we identify the groups of platelets that are connected to one another.

### Percolating cluster analysis

To take into account the anisotropic arrangement of the apatite mineral within the MCF, we investigated percolation paths developed in the three CS directions, i.e. W, T, L axes.

The percolating cluster is determined by the following algorithm:

- For each cluster we individuate the vertices of its platelets.
- To determine percolation paths in the equatorial plane, we select the clusters from step (a) with vertices of the platelets that are outside a circumference of diameter equal to 90% of the MCF diameter.
  1. For each selected cluster we determine the minimum (w_min_) and maximum (w_max_) coordinates of the vertices along W direction and compute the distance d_W_:
    4 $$ d_{W} = w_{\max } - w_{\min } $$
    In order to take into account the cylindrical geometry of the simulation domain, in W direction we assume that the clusters are percolating if their length d_W_ is greater or equal to the chord length W_percolation_:
    5 $$ d_{W} \ge W_{percolation} $$
    In post processing analysis, we observed that a bistable behaviour^40,60^ is developed when considering clusters composed by more than four platelets. Thus, we identified a minimum chord length of 50 nm and 120 nm for the MCF diameter of 50 nm and 200 nm respectively, for which a well-defined sigmoidal trend for the percolation probability is achieved.
  2. For each selected cluster we identified the minimum (t_min_) and maximum (t_max_) coordinates of the vertices along T direction and compute the distance d_T_:
    6 $$ d_{T} = t_{\max } - t_{\min } $$
    Analogously to the analysis performed for spanning clusters in W direction, we consider that clusters are percolating in T direction if the following relation is valid:
    7 $$ d_{T} \ge T_{percolation} $$
    In post-processing analysis, we observed that the system has a percolation-like behaviour^40,60^ when the minimum chord length T_percolation_ is equal to 25 nm and 50 nm for the MCF diameter of 50 nm and 200 nm, respectively.
- For percolation paths in the longitudinal direction, we selected the clusters from step (a) with coordinates of vertices along L direction that are outside a cylinder of length equal to 950 nm, i.e. 95% length of the MCF.
- In the longitudinal direction we observed that a bistable behaviour of the system is achieved when we analysed clusters that could span a MCF with reduced length according to recent experimental tomographic data^2^. Therefore, we considered for each cluster identified in step (a) the minimum (*ℓ*_min_) and maximum (*ℓ*_max_) coordinates of the vertices along the L direction. We compute the distance:
  8 $$ d_{L} = \ell_{\max } - \ell_{\min } $$
  and selected the clusters that verify the condition:
  9 $$ d_{L} \ge L{}_{cluster} $$
  where L_cluster_ is equal to 300 nm and 450 nm for the MCF diameter of 50 nm and 200 nm respectively. These thresholds are frequent values of MCF length individuated experimentally in^2^.

### Percolation probability

The percolation probability for a given VF is obtained from Eq. (10):

10 $$ P(VF) = \frac{{n_{p} }}{N} $$

where n_p_ is the number of configurations characterized by percolating clusters and N is the number of realizations assessed by means of the cluster analysis.

The resulting probabilities were plotted in function of the volume fraction and fitted with the hyperbolic tangent function^40^, i.e.:

11 $$ P(VF) = \frac{1}{2} \cdot \left[ {1 + \tanh \left( {\frac{{VF - VF_{c} }}{\Delta }} \right)} \right] $$

where VF_c_ is the critical VF and represents the VF at which the percolation probability is equal to 0.5 and Δ characterizes the width of the percolation transition.

## Supplementary Information

Supplementary Information.

## Data Availability

All data generated or analysed during this study are included in this published article (and its Supplementary Information files).
