# Supplementary material for: Percolation networks inside 3D model of the mineralized collagen fibril
Source: Sci Rep. 2021 May 31;11:11398. doi: 10.1038/s41598-021-90916-x (PMC8166932; doi:10.1038/s41598-021-90916-x)
Supplement: Supplementary file 1 — Supplementary Information. [file 41598_2021_90916_MOESM1_ESM.docx]

Supplementary Information

Percolation Networks inside

3D Model of the Mineralized Collagen Fibril

Fabiano Bini^1^*, Andrada Pica^1^, Andrea Marinozzi^2^, Franco Marinozzi^1^

^1^Department of Mechanical and Aerospace Engineering, “Sapienza” University of Rome, via Eudossiana, 18 - 00184, Rome, Italy.

^2^Orthopedy and Traumatology Area, “Campus Bio-Medico” University, via Alvaro del Portillo, 200 - 00128, Rome, Italy.

**S1 Model parameters**

The mineral volume fraction (VF) is calculated according to Eq. (S1):

|  | (S1) |
| --- | --- |

where *ℓ*, w, t are the length, width and thickness of the mineral platelet, a_L_, a_W_ and a_T_ are the distances between the platelets in the longitudinal, width and thickness direction. We consider all random values for the geometrical parameters that describe the apatite mineral. These values are obtained by means of random extraction from Gaussian Probability Density Functions in the ranges indicated in Literature [1-4]. In Table S1 we report the average dimensions of the apatite crystals for each degree of mineralization and for each MCF diameter.

**Table S1.** Average dimensions of the apatite platelets and average distances between the mineral crystals for each degree of mineralization and for each MCF diameter. For the MCF with 50 nm diameter we considered 136 platelets, while the MCF with 200 nm diameter is characterized by 526 platelets.

|  | MCF diameter = 50 nm | | | | | | MCF diameter = 200 nm | | | | | |
| --- | --- | --- | --- | --- | --- | --- | --- | --- | --- | --- | --- | --- |
| VF | w  (nm) | t  (nm) | *ℓ*  (nm) | a_W_  (nm) | a_T_  (nm) | a_L_  (nm) | w  (nm) | t  (nm) | *ℓ*  (nm) | a_W_  (nm) | a_T_  (nm) | a_L_  (nm) |
| 7% | 7.69 | 2.10 | 43.90 | 6.31 | 3.29 | 90.1 | 14.47 | 3.00 | 71.5 | 40.53 | 2.89 | 62.49 |
| 12% | 7.71 | 2.68 | 59.30 | 6.28 | 2.72 | 74.69 | 21.78 | 3.15 | 77.12 | 33.22 | 2.75 | 56.88 |
| 17% | 8.48 | 2.93 | 69.97 | 5.51 | 2.47 | 64.03 | 27.58 | 3.27 | 82.97 | 27.41 | 2.63 | 51.02 |
| 22% | 9.32 | 3.10 | 78.00 | 4.67 | 2.30 | 56.00 | 32.80 | 3.38 | 87.07 | 22.19 | 2.52 | 46.93 |
| 27% | 9.90 | 3.23 | 86.53 | 4.09 | 2.16 | 47.47 | 37.64 | 3.45 | 91.20 | 17.36 | 2.44 | 42.79 |
| 32% | 10.52 | 3.41 | 91.55 | 3.48 | 1.99 | 42.45 | 41.80 | 3.55 | 94.51 | 13.19 | 2.35 | 39.49 |
| 37% | 11.18 | 3.47 | 97.77 | 2.82 | 1.93 | 36.23 | 43.14 | 3.67 | 102.15 | 11.86 | 2.22 | 31.85 |
| 42% | 11.72 | 3.61 | 101.62 | 2.28 | 1.79 | 32.38 | 44.41 | 3.85 | 107.57 | 10.59 | 2.05 | 26.43 |
| 47% | 12.00 | 3.75 | 106.79 | 1.99 | 1.65 | 27.21 | 45.80 | 3.97 | 112.84 | 9.19 | 1.92 | 21.15 |
| 52% | 12.48 | 3.82 | 111.49 | 1.52 | 1.58 | 22.5 | 47.85 | 4.07 | 116.27 | 7.14 | 1.83 | 17.73 |

**S2 Initial configuration**

For each mineral content of the MCF, we used the Metropolis algorithm [25] to generate mineral configurations. We considered that, initially, the centroids of the apatite platelets are placed in correspondence of the sites of a staggered prismatic lattice within the cylinder (Fig. S1). In Literature [2, 5], there is limited evidence with regard to the arrangement of apatite platelets within the MCF. We developed the geometry of the staggered prismatic lattice following the model of apatite organization proposed by [5]. The staggered pattern is obtained by considering a periodic length of 67 nm between adjacent sites in longitudinal direction [5]. In the equatorial plane, the distance between the lattice sites is set so that the platelets with highest VF may be incorporated within the MCF. Considering the range indicated in Literature concerning mineral dimensions and apatite crystals interdistance [2, 3], we assume the hypothesis that the maximum number of aligned platelets to the coordinate system (CS) that may be within the MCF diameter along the width direction is equal to three. Analogously, for the T direction we assumed that the number of aligned platelets with the CS that may be incorporated within the MCF diameter of 50 nm and 200 nm is maximum 8 and 32, respectively.

Hence, in compliance with the geometric constraints, the total number of sites is 136 for the MCF with 50 nm diameter and 526 for the MCF with 200 nm diameter.


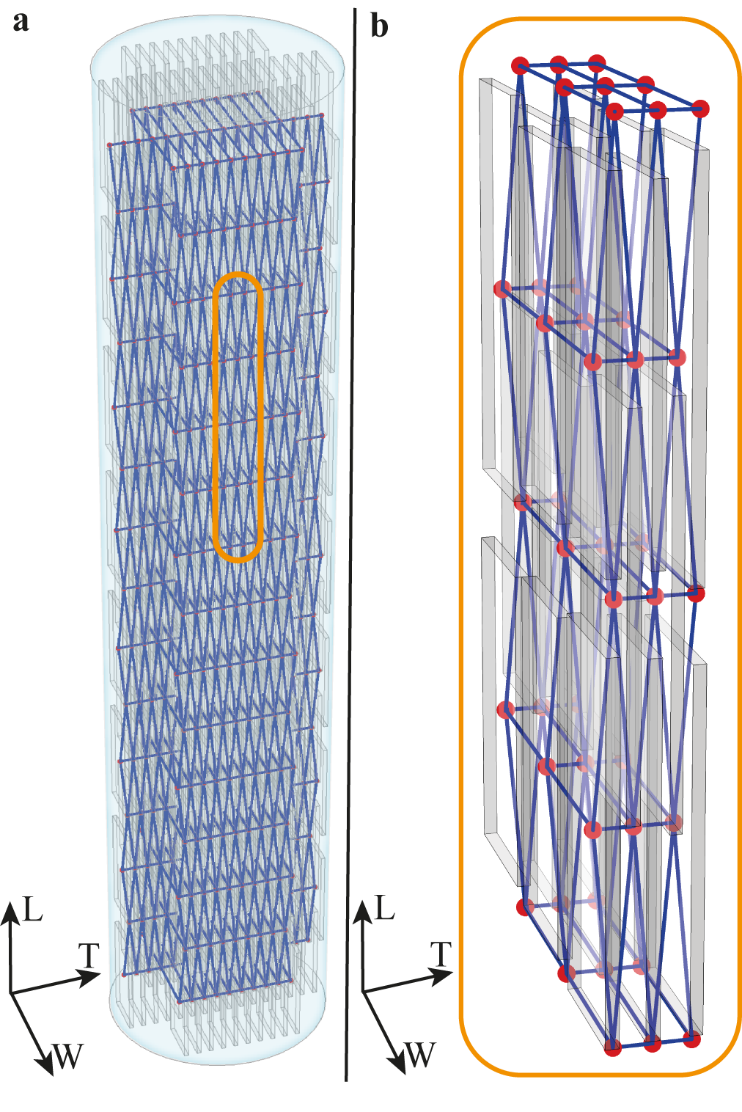


**Fig. S1.** Representation of the starting configuration for the 3D apatite model (light grey) on the staggered prismatic lattice (dark blue). The centroids (red dots) of apatite platelets are placed in correspondence of the lattice sites (b).

**S3 Interpenetration Algorithm**

The interpenetration detection algorithm can be summarised by the following steps:

1. Consider that a platelet A_i_ is randomly displaced and oriented within the MCF and its centroid is identified by the coordinates (wi_c, ti_c, ℓi_c). To speed up the algorithm [6], we consider an exploring volume centred in correspondence of the A_i_ platelet centroid and aligned with the CS. We assume the hypothesis that the dimension of the exploring volume along the W axis is equal to the MCF diameter, along T axis is ten times the average thickness of apatite platelets, i.e. T_average_ = 3.5 nm [2, 5], and along L axis is equal to 30 percent of the MCF length. We apply the interpenetration detection algorithm to the platelets whose centroids belong to the exploring volume.
2. We calculate the nearest point (p_j_) of each platelet within the exploring volume (e.g. platelet A_j_) to the centroid of the platelet A_i_. The point p_j_ is obtained by means of a gradient based method that minimizes the Euclidean norm between the two points:

|  | (S2) |
| --- | --- |

where (w_j_, t_j_, *ℓ*_j_) are the coordinates of the nearest point of the platelet A_j_ to the centroid of the platelet A_i_.

1. We calculate the projection of the distance between the centroid of the platelet A_i_ and the point p_j_ identified at step (b) along the three axes of the CS:

| $\Delta d_{W}=\left\vert w_{c\_i}-w_{p\_j}\right\vert$ | (S3) |
| --- | --- |
| $\Delta d_{T}=\left\vert t_{c\_i}-t_{p\_j}\right\vert$ | (S4) |
|  | (S5) |

1. If the following conditions are simultaneously meet, an interpenetration between A_i_ and A_j_ occurs for more than 10 percent of A_i_ volume and the move is rejected:

|  | (S6) |
| --- | --- |

1. Platelets can interpenetrate when two conditions of Equation S6 are verified. For these scenarios, we analysed the spatial positions of vertices of platelets A_i_ and A_j_. We made the hypothesis that the edges of platelet A_j_ that interpenetrate the platelet A_i_ could not cross the whole crystal thickness. Therefore, we reject the move if one of conditions S7a-b, S8a-d are met. Assuming that vertex m indicates a vertex of the lower base of the platelets and vertex n indicates a vertex of the upper base of the platelets, the relations S7a and S7b are expressed as follows:

| $\left\{\begin{matrix} \text{t} \text{component} \text{of} \text{vertex} \text{m} \text{of} \text{platelet} \text{A}_{\text{j}}\text{<t} \text{component} \text{of} \text{vertex} \text{m} \text{of} \text{platelet} \text{A}_{\text{i}} \\ \text{t} \text{component} \text{of} \text{vertex} \text{n} \text{of} \text{platelet} \text{A}_{\text{j}}\text{>t} \text{component} \text{of} \text{vertex} \text{n} \text{of} \text{platelet} \text{A}_{\text{i}} \end{matrix}\right.$ | (S7a) |
| --- | --- |
| $\left\{\begin{matrix} \text{t} \text{component} \text{of} \text{vertex} \text{m} \text{of} \text{platelet} \text{A}_{\text{j}}\text{>t} \text{component} \text{of} \text{vertex} \text{m} \text{of} \text{platelet} \text{A}_{\text{i}} \\ \text{t} \text{component} \text{of} \text{vertex} \text{n} \text{of} \text{platelet} \text{A}_{\text{j}}\text{<t} \text{component} \text{of} \text{vertex} \text{n} \text{of} \text{platelet} \text{A}_{\text{i}} \end{matrix}\right.$ | (S7b) |
| $\text{Number} \text{of} \text{vertices} \text{of} \text{A}_{\text{i}} \text{lower} \text{base} \text{that} \text{are} \text{internal} \text{to} \text{platelet} \text{A}_{\text{j}}\text{>2}$ | (S8a) |
| $\text{Number} \text{of} \text{vertices} \text{of} \text{A}_{\text{i}} \text{upper} \text{base} \text{that} \text{are} \text{internal} \text{to} \text{platelet} \text{A}_{\text{j}}\text{>2}$ | (S8b) |
| $\text{Number} \text{of} \text{vertices} \text{of} \text{A}_{\text{j}} \text{lower} \text{base} \text{that} \text{are} \text{internal} \text{to} \text{platelet} \text{A}_{\text{i}}\text{>2}$ | (S8c) |
| $\text{Number} \text{of} \text{vertices} \text{of} \text{A}_{\text{j}} \text{upper} \text{base} \text{that} \text{are} \text{internal} \text{to} \text{platelet} \text{A}_{\text{i}}\text{>2}$ | (S8d) |

1. If none of the above conditions is verified, the attempted move and rotation are accepted.

**S4 Equilibrium assessment**

Equilibrium is monitored by computing average measures as Radial Distribution Function (RDF) and the nematic order parameter (S).

The radial distribution function g_2_(r) is a powerful tool used to analyse the complex 3D platelets position. The RDF is obtained as a normalization of the local density of platelets at a given distance **r** from a given reference platelet to the overall mineral crystal density in the volume.

The platelets density (ρ) in the cylindric volume is obtained from Eq. S9 as the ratio between the number of platelets in the MCF, i.e. n_HA_, and the MCF volume, i.e. V_MCF_.

| $\rho=\frac{n_{HA}}{V_{MCF}}$ | (S9) |
| --- | --- |

The local density is determined considering the number of platelets in concentric shells with finite thickness Δr that are at a distance comprises between $\left(r-\frac{\Delta r}{2},r+\frac{\Delta r}{2}\right)$from the reference platelet divided by the shell volume. In order to take into account the cylindrical geometry of the MCF, we considered concentric spherical shells in the equatorial plane and elliptic shells in the longitudinal direction (Fig. S2).

In the equatorial plane, the volume of the spherical shell of thickness Δr is obtained as follows:

| $V_{shell EQ}\left(r,\Delta r\right)=\frac{4}{3}\cdot\pi\cdot\left[\left(r+\frac{\Delta r}{2}\right)^{3}-\left(r-\frac{\Delta r}{2}\right)^{3}\right]$ | (S10) |
| --- | --- |


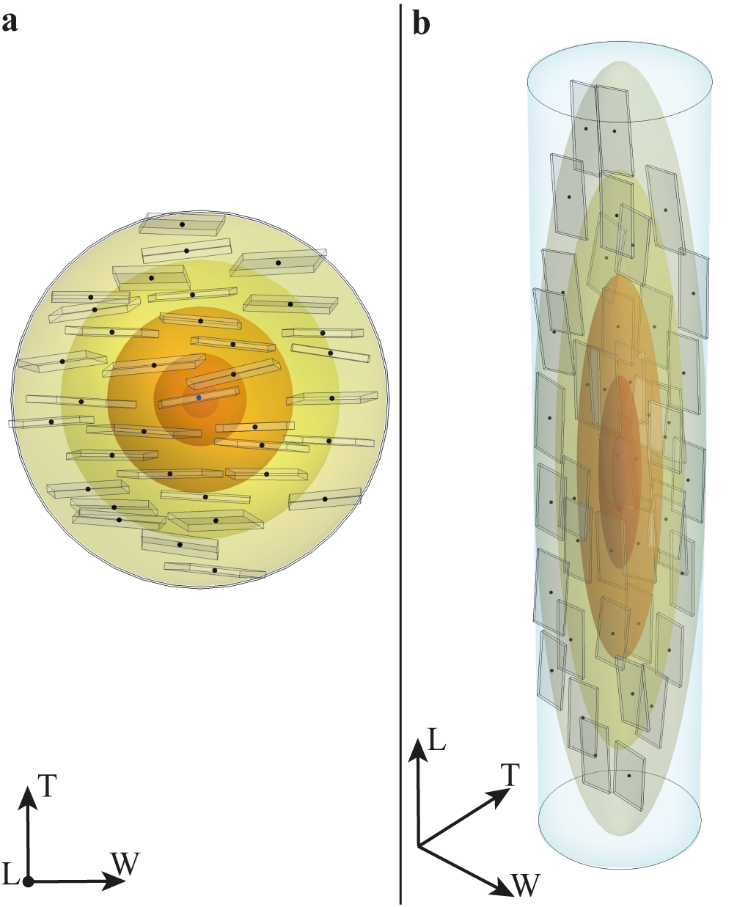


**Fig. S2.** Spherical (a) and ellipsoidal (b) shells used in the sampling for the radial distribution function in the equatorial plane and longitudinal direction, respectively. The black points represent the centroids of the platelets (light grey) at equilibrium within the MCF characterized by a diameter of 200 nm.

In the longitudinal direction we consider an elliptic shell with thickness Δr_W_ in W direction, Δr_T_ in T direction and Δr_L_ in L direction. The shell volume is achieved as:

| $V_{shell L}\left(r,\Delta r\right)=\frac{4}{3}\cdot\pi\cdot\left[\left(r_{W}+\frac{\Delta r_{W}}{2}\right)\cdot\left(r_{T}+\frac{\Delta r_{T}}{2}\right)\cdot\left(r_{L}+\frac{\Delta r_{L}}{2}\right)-\left(r_{W}-\frac{\Delta r_{W}}{2}\right)\cdot\left(r_{T}-\frac{\Delta r_{T}}{2}\right)\cdot\left(r_{L}-\frac{\Delta r_{L}}{2}\right)\right]$ | (S11) |
| --- | --- |

The largest shell radius is limited in the equatorial plane to half MCF diameter and in the longitudinal direction to half MCF length.

For a given mineral VF, we compute the distance between the centroids of all pair of mineral platelets from all of the generated configurations. Periodic boundary conditions are implemented in the three spatial directions.

The pairwise separations are then sorted into a histogram in which each bin has width Δr. The average number of platelets, i.e. n(r), whose distance from a given reference platelet lies in the interval $\left(r-\frac{\Delta r}{2},r+\frac{\Delta r}{2}\right)$ is given by:

| $n(r)=\frac{n_{k}(r)}{M\cdot n_{HA}}$ | (S12) |
| --- | --- |

where n_k_(r) represents the number of platelets contained in the bin k of the histogram corresponding to the distance **r** and M is the total number of configurations.

Therefore, the RDF for a particular value of **r** is obtained from Eq. S13:

| $g_{2}(r)=\frac{1}{\rho}\cdot\frac{n(r)}{V_{shell}(r)}$ | (S13) |
| --- | --- |

RDF is expected to reach constant values at the equilibrium. For instance, in Figure S3 we present the RDF plots for a mineral VF of 47 percent within the MCF of 50 nm and 200 nm diameter, respectively. We assumed that the system has reached the equilibrium when the g_2_(r), for large distances approaches 1 [6], specifically it is in a range (0.9;1.1). We identified this condition after overall 2.1·10^6^ moves and rotations.


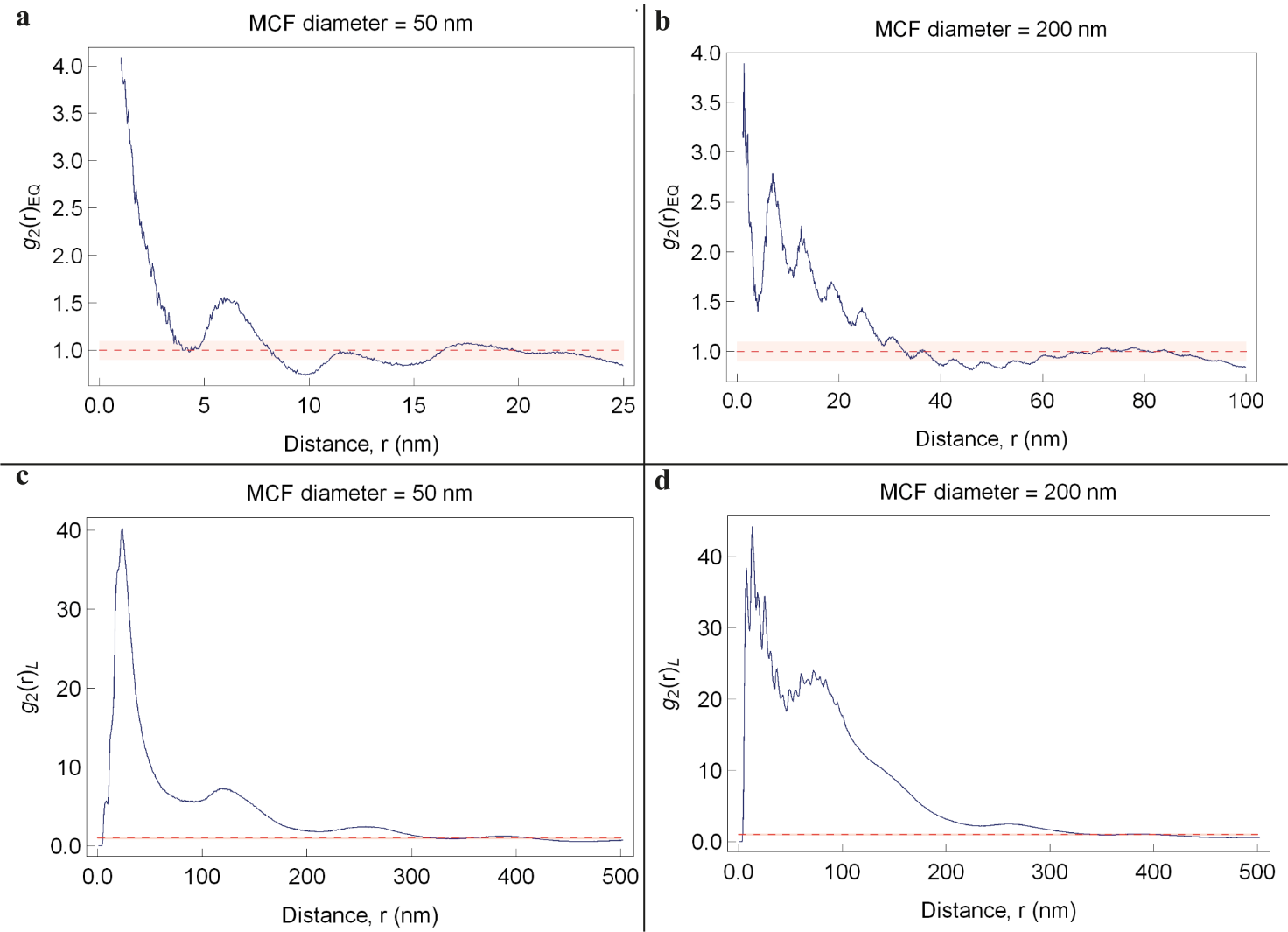


**Fig. S3.** Radial distribution function g_2_(r) versus the interplatelets distance r in the equatorial plane (a, b) and in longitudinal direction (c, d) for an equilibrium configuration of platelets at 47 percent mineral VF within the MCF characterized by a diameter of 50 nm (a, c) and 200 nm (b, d).

For a given realization, the nematic order parameter S is obtained as the maximum eigenvalue of the tensor **Q** defined as follows [7]:

| $Q=\frac{1}{n_{HA}}\cdot\sum\frac{3}{2}\cdot u_{\alpha}\cdot u_{\beta}-\frac{1}{2}I$ | (S14) |
| --- | --- |

where n_HA_ is the number of platelets within the fibril, u_α_ u_β_ are the component of the unit vector **u** parallel to the longitudinal axis of the platelet, with α and β = L, W, T are indices referring to the absolute coordinate system and **I** is the identity matrix.

The nematic order parameter provides information about the alignment of apatite platelets. In a fully aligned configuration S is unity, while in an isotropic case S is zero. In this case, after roughly 2.1·10^6^ moves and rotations, i.e. the equilibrium state identified by the RDF, S remains in a narrow range (0.98; 1) reflecting the morphological constraint of roughly aligned platelets to the c-axis of the collagen fibril [8] (Fig. S4).


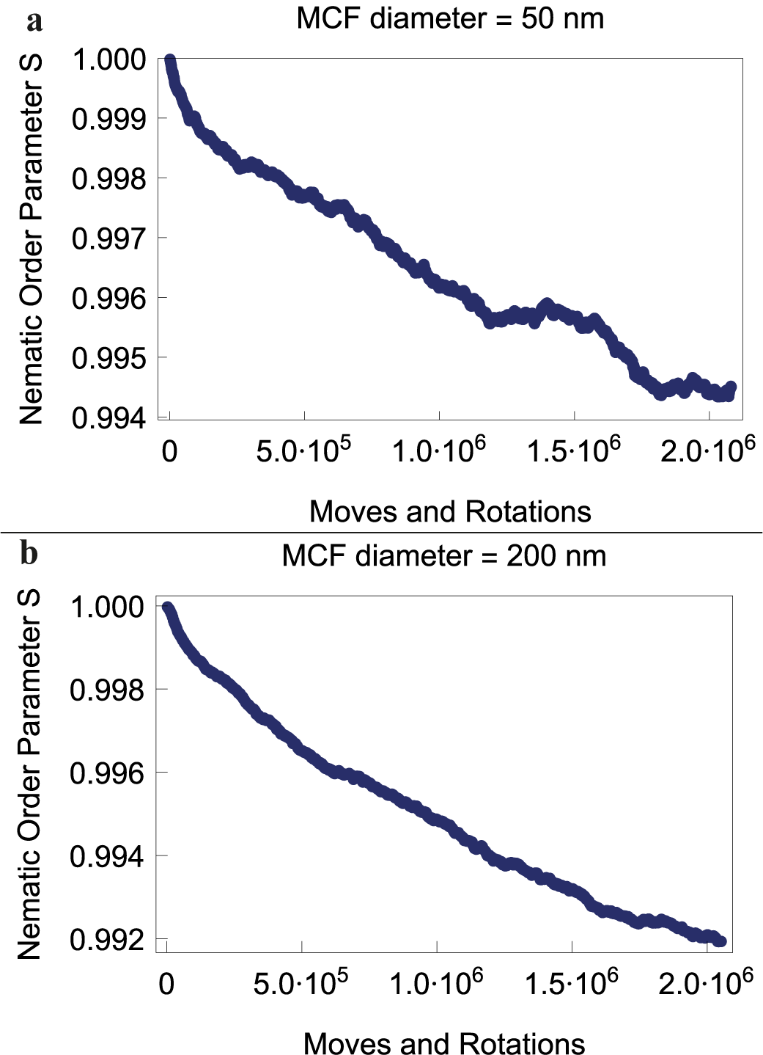


**Fig. S4.** Nematic order parameter calculated for mineral volume fraction of 47 percent within the mineralized collagen fibril with diameter of 50 nm (a) and 200 nm (b).

**S5 Influence of connectivity distance**

The structure of the network depends crucially on the connectivity distance considered (Fig. S5). The translation of the critical VF to lower mineral content for increasing values of δ as observed in Fig. 2 of the main text is also predicted by an analytical relation [7], which assumes that the critical VF, i.e. VF_c_, is proportional to the ratio of the platelet volume V_p_ and the connection volume V_connect_ of two platelets:

|  | (S15) |
| --- | --- |

where w, t, *ℓ* are the dimensions of crystal platelet,

|  | (S16) |
| --- | --- |
|  | (S17) |


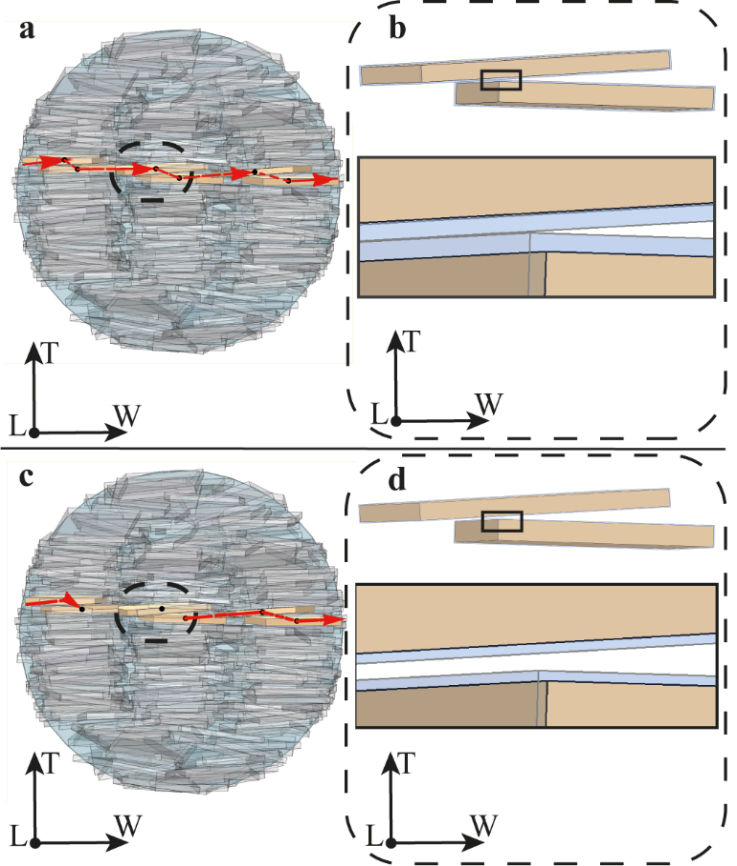


**Fig. S5.** Schematic example of the influence of connectivity threshold on the formation of percolating clusters. In (a) we show a group of platelets that are connected by means of their hydrated layers. A spanning cluster in W direction is formed since the thickness of the shell (light blue) that covers the platelets is such to enable the connection between platelets (b). In (c) the same platelets illustrated in (a) form multiple, smaller and not percolating clusters if a reduced shell thickness is considered (d). In (a) and (c) the black points represent the platelets centroids and the dotted arrows connecting two points are assigned when the corresponding platelets are connected. The example is achieved from a configuration of apatite mineral at 47 percent of mineral volume fraction within MCF of 200 nm diameter.

**Supplementary References**

1. W. Wagermaier, K. Klaushofer, P. Fratzl. Fragility of Bone Material Controlled by Internal Interfaces. Calcif Tissue Int 97, 201–212 (2015). https://doi.org/10.1007/s00223-015-9978-4
2. M.A. Rubin, I. Jasiuk, J. Taylor, J. Rubin, T. Ganey, R.P. Apkarian. TEM analysis of the nanostructure of normal and osteoporotic human trabecular bone. Bone 33, 270–282 (2003). https://doi.org/10.1016/S8756-3282(03)00194-7
3. F. Bini, A. Pica, A. Marinozzi, F. Marinozzi. A 3D Model of the Effect of Tortuosity and Constrictivity on the Diffusion in Mineralized Collagen Fibril. Sci Rep 9, 2658 (2019). https://doi.org/10.1038/s41598-019-39297-w
4. S. Weiner, P.A. Price. Disaggregation of bone into crystals. Calcif Tissue Int 39 (1986), 365-375. https://doi.org/10.1007/BF02555173
5. I. Jäger, P. Fratzl Mineralized collagen fibrils: a mechanical model with a staggered arrangement of mineral particles. Biophys. J. 79, 1737–1746 (2000). https://doi.org/10.1016/S0006-3495(00)76426-5
6. S. Torquato. Random heterogeneous materials: microstructure and macroscopic properties, Springer Science, New York, 2002.
7. M. Mathew, T. Schilling, M. Oettel. Connectivity percolation in suspensions of hard platelets. Phys. Rev. E 85 (2012), 061407. https://doi.org/10.1103/PhysRevE.85.061407
8. M. Georgiadis, R. Müller, P. Schneider, J. R. Techniques to assess bone ultrastructure organization: orientation and arrangement of mineralized collagen fibrils. J. R. Soc. Interface 13, 20160088 (2016). https://doi.org/10.1098/rsif.2016.0088
